# An analytical model for computing the sound power of an unbraced irregular-shaped plate of variable thickness

**DOI:** 10.1038/s41598-018-33645-y

**Published:** 2018-10-18

**Authors:** Meng Koon Lee, Mohammad Hosseini Fouladi, Satesh Narayana Namasivayam

**Affiliations:** School of Engineering, Taylor’s University, No.1 Jalan Taylor’s, 47500 Subang Jaya, Selangor Malaysia

## Abstract

An irregular-shaped plate with dimensions identical to a guitar soundboard is chosen for this study. It is well known that the classical guitar soundboard is a major contributor to acoustic radiation at high frequencies when compared to the bridge and sound hole. This paper focuses on using an analytical model to compute the sound power of an unbraced irregular-shaped plate of variable thickness up to frequencies of 5 kHz. The analytical model is an equivalent thin rectangular plate of variable thickness. Sound power of an irregular-shaped plate of variable thickness and with dimensions of an unbraced Torres’ soundboard is determined from computer analysis using ANSYS. The number of acoustic elements used in ANSYS for accurate simulation is six elements per wavelength. Here we show that the analytical model can be used to compute sound power of an unbraced irregular-shaped plate of variable thickness.

## Introduction

There are many acoustic applications which use irregular-shaped plates. Here, we consider a specific irregular-shaped plate having the form and dimensions of the Torres’ soundboard of variable thickness. This soundboard is found in some classical guitars.

It is well known that from 300 Hz to 5 kHz, the surface of the soundboard is the most important frequency dependent radiation area of the classical guitar whilst from 70 Hz to 300 Hz the sound hole is most important^1^. However, in the study of acoustic radiation^2^, there is lack of information beyond 500 Hz. Numerous discrete models of guitars using 2-, 3- and 4-degrees of freedom have been proposed to study their frequency response up to 250 Hz^3–5^. Assessment of frequency response and acoustics of soundboard up to 5 kHz requires a continuum model^6^ using the theory of thin plates^7,8^. This theory can also be applied to an irregular-shaped plate having the shape of the soundboard^9^.

This type of irregular-shaped plate can be structurally designed to withstand the cumulative string tension of the first, second and third nylon strings and the fourth, fifth and sixth wire-wound nylon strings under playing conditions. Since a guitar soundboard can be considered as a modified form of a rectangular plate^10^, this paper will use the complete analytical solution of an equivalent rectangular plate^11^ to compute the sound power of an unbraced irregular-shaped plate which is identical in shape to that of the guitar soundboard.

It has been determined^12^ that the frequencies of a classical guitar can vary from 70 Hz to just under 2 kHz. It is also capable of producing harmonic notes. To account for these higher frequencies it was suggested that the frequency range be extended to 20 kHz^13^, which is the upper threshold of human hearing. For practical purposes, it is proposed to limit this range from 70 Hz to 5 kHz as this is within the critical band of hearing^14–16^. Also, ISO R226, 1961; ISO 226, 1987 indicates that the lower threshold of human hearing (1 kHz to 5 kHz) is within this range^17,18^. Sound power for the unbraced irregular-shaped plate is computed within this frequency range.

## Results

**Figure 1:**
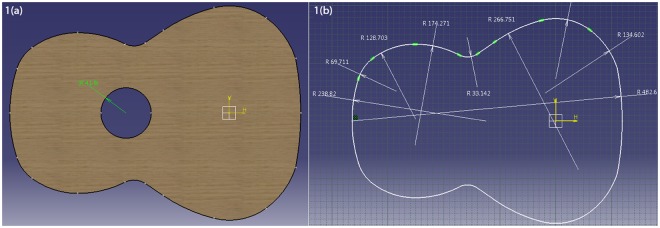
Dimensions of an irregular-shaped plate. (**a**) Radius of sound hole (mm). Area of plate = 0.125 m^2^. (**b**) Dimensions of contour of plate (mm).

An irregular-shaped plate having the form and dimensions of a Torres’ soundboard of a constant thickness used in this study is constructed using a 9-centre method^19^. The dimensions of the sound hole and those of the contour of the plate are shown in Fig. 1(a,b) respectively. This plate has a constant thickness of 2 mm.

### Similarity of mode shapes

**Figure 2:**
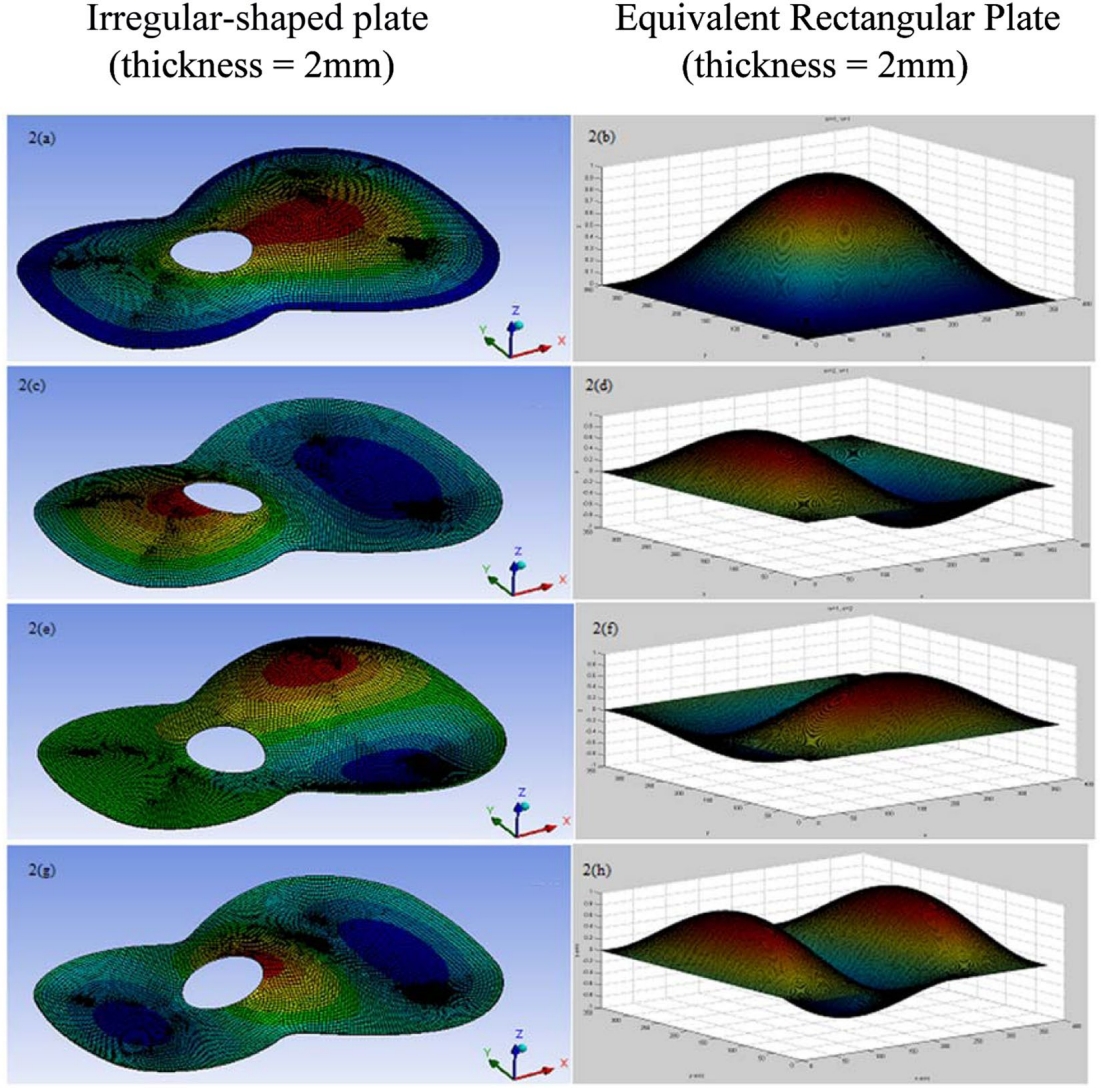
Comparison of mode shapes between irregular-shaped plate and the equivalent rectangular plate for the lowest 4 modes^11^. (**a**) Mode 1:T(1,1), (**b**) Mode 1: *m* = 1*, n* = 1, (**c**) Mode 2: T(1,2), (**d**) Mode 2: *m* = 2, *n* = 1, (**e**) Mode 3: T(2,1), (**f**) Mode 3: *m* = 1*, n* = 2, (**g**) Mode 4: T(1,3), (**h**) Mode 4: *m* = *3, n* = 1.

The similarity of mode shapes between those of the irregular-shaped plate and an equivalent solid rectangular plate are shown in Fig. 2. Mode shapes of the equivalent rectangular plate are computed using a MATLAB program for the simply supported boundary condition whilst those of the irregular-shaped plate are simulated using “displacement” boundary condition as the simply supported condition is not supported for solid bodies in ANSYS^20^. The notation for modes of the irregular-shaped plate follows those for a guitar soundboard^21^ is shown in Fig. 2(a,c,e and g).

### Equivalent rectangular plate

To date, there is no standard code of practice for equivalent rectangular plate in the analysis of soundboards of musical instruments unlike for example, ASME codes for equivalent solid rectangular plates in place of perforated plates with triangular and rectangular patterns, Myung and Jong^22^.

As stated in^22^, the ASME code for equivalent solid rectangular plates instead of original perforated plates does not apply to modal analysis. Therefore, an expression for the equivalent material property such as Young’s modulus as a function of ligament efficiency was derived by Myung and Jong^22^ in attempts to obtain natural frequencies of equivalent solid rectangular plates that are closest to those of the original perforated plates. An irregular-shaped plate identical in shape to that of a guitar soundboard has only one perforation called the sound hole. Therefore, the ligament ratio concept for perforated plates^22^ cannot be applied in this study.

It is therefore proposed to adopt some criteria to determine the dimensions of an equivalent rectangular plate of constant thickness. The criteria for determining its dimensions^11^ are as follows:

- The rectangular plate vibrates transversely under dynamic compression in the direction of its longitudinal grain. The longitudinal Young’s modulus is identical to that of the guitar soundboard material as its longitudinal grain is always aligned parallel to the strings and the guitar soundboard is constantly under dynamic compression under playing conditions.
- The area and its thickness are the same as those of the irregular-shaped plate without the hole (area = 0.131 m^2^, thickness = 2 mm),
- An aspect ratio close to but not equal to unity gives best results for natural frequencies as shown in Table 1. An aspect ratio equal to unity would result in many degenerate modes and therefore not representative of the original situation, and

**Table 1:** Comparison of natural frequencies for variable thickness plates.

Mode No:	ANSYS (irregular-shaped plate of Fig. 1)	*PS3 (rectangular plate) 374.3 mm × 350.0 mm	% error of PS3 rel. to ANSYS
	α = 0.154 degrees		
	Frequency, *f* (Hz)		
1	88.53	73.96	−16
2	140.16	177.46	26
3	236.22	192.34	−18
4	249.57	295.83	18
5	344.21	349.97	1
6	413.81	389.62	−5
7	444.96	468.34	5
8	456.21	493.12	8
9	563.58	591.47	4
10	594.85	665.62	11
11	715.58	665.82	−6
12	730.73	709.84	−2
13	747.94	769.32	2
14	823.84	907.13	10
15	836.42	941.83	12
nodes	13978		
elements	1924
- The mode shapes of the equivalent solid rectangular plate and those of the irregular-shaped plate are similar.

The above criteria resulted in minimal errors in the computed natural frequencies relative to those obtained from ANSYS. Based on the above criteria, choosing a *y*-value of 0.35 m for a rectangular plate resulted in an *x*-value of 0.3743 m. The aspect ratio of 0.935 is close to but not equal to unity. Thus, the dimensions of the equivalent rectangular plate are *a* = 374.3 mm, *b* = 350 mm.

These criteria are extended to determine the natural frequencies of an equivalent rectangular plate of variable thickness by multiplying the corresponding results for equivalent rectangular plate of constant thickness by a factor of (1 + α) as the mode shapes of these two plates for the simply supported boundary conditions are used to determine natural frequencies. Mode shapes for the constant and variable thickness plates are shown in Equations (A1.5) and (A1.6) of “Supplementary Information”.

### Description of thin plates of constant thickness

The flexural deflection of the plate^8^ without damping is defined by:

1a $$\rho {h}_{0}\frac{{\partial }^{2}w}{\partial {t}^{2}}+{D}_{0}(\frac{{\partial }^{4}w}{\partial {x}^{4}}+2\frac{{\partial }^{4}w}{\partial {x}^{2}\partial {y}^{2}}+\frac{{\partial }^{4}w}{\partial {y}^{4}})=f(x,y,t)$$

1b $${\rm{or}}\,\frac{{\partial }^{2}w}{\partial {t}^{2}}+\frac{{D}_{0}}{\rho {h}_{0}}(\frac{{\partial }^{4}w}{{\partial }^{4}x}+2\frac{{\partial }^{4}w}{\partial {x}^{2}\partial {y}^{2}}+\frac{{\partial }^{4}w}{{\partial }^{4}y})=\frac{f(x,y,t)}{\rho {h}_{0}}$$

where $${D}_{0}=\frac{E{h}_{0}^{3}}{12(1-{\nu }^{2})}$$

- *f*(*x*, *y*, *t*) = external forcing function,
- *h*_0_ = thickness of plate,
- *ρ* = density of plate material (kg/m^3^), and
- ν = Poisson’s ratio of plate material.

Equation (1b) is a 4^th^ order parabolic partial differential equation with constant coefficients. Its version for plates with variable thickness is as shown the following section.

### Description of thin plates of variable thickness

For an undamped plate, its flexural deflection^8^ is given by:

2 $$\begin{array}{ll}\rho h(x,y)\tfrac{{\partial }^{2}w}{\partial {t}^{2}} & +D(\tfrac{{\partial }^{4}w}{\partial {x}^{4}}+2\tfrac{{\partial }^{4}w}{\partial {x}^{2}\partial {y}^{2}}+\tfrac{{\partial }^{4}w}{\partial {y}^{4}})+2\tfrac{\partial D}{\partial x}\tfrac{\partial }{\partial x}(\tfrac{{\partial }^{2}w}{\partial {x}^{2}}+\tfrac{{\partial }^{2}w}{\partial {y}^{2}})+2\tfrac{\partial D}{\partial y}\tfrac{\partial }{\partial y}(\tfrac{{\partial }^{2}w}{\partial {x}^{2}}+\tfrac{{\partial }^{2}w}{\partial {y}^{2}})\\  & +\,\tfrac{{\partial }^{2}D}{\partial {x}^{2}}(\tfrac{{\partial }^{2}w}{\partial {x}^{2}}+\nu \tfrac{{\partial }^{2}w}{\partial {y}^{2}})+\tfrac{{\partial }^{2}D}{\partial {y}^{2}}(\tfrac{{\partial }^{2}w}{\partial {y}^{2}}+\nu \tfrac{{\partial }^{2}w}{\partial {x}^{2}})+2(1-\nu )\tfrac{{\partial }^{2}w}{\partial x\partial y}(\tfrac{{\partial }^{2}D}{\partial x\partial y})=f(x,y,t)\end{array}$$

**Figure 3:**
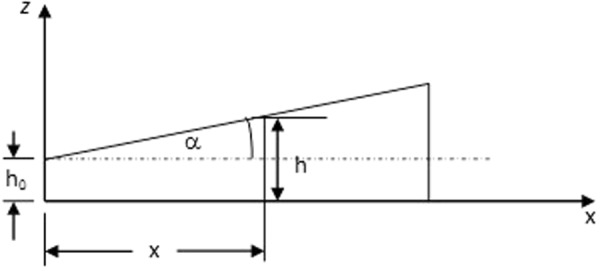
Variation of thickness in *x*-direction.

Assume that the thickness of a rectangular plate be given by:

3 $$h={h}_{0}[1+\alpha (x/{h}_{0})]$$

where *h*_0_, *α* and *x* are as shown in Fig. 3.

Substituting Equation (3) into the flexural stiffness of the plate given by $D=\frac{E{h}^{3}}{12(1-{\nu }^{2})}$, we get:

4 $$D=\frac{E{h}_{0}^{3}{(1+\alpha \frac{x}{{h}_{0}})}^{3}}{12(1-{\nu }^{2})}$$

Equation (2) can then be re-written as:

5 $$\begin{array}{l}\tfrac{{\partial }^{2}w}{\partial {t}^{2}}+(\tfrac{C}{12}){(1+\alpha \tfrac{x}{{h}_{0}})}^{2}(\tfrac{{\partial }^{4}w}{\partial {x}^{4}}+2\tfrac{{\partial }^{4}w}{\partial {x}^{2}\partial {y}^{2}}+\tfrac{{\partial }^{4}w}{\partial {y}^{4}})+(\tfrac{C}{2})(\tfrac{\alpha }{{h}_{0}})(1+\alpha \tfrac{x}{{h}_{0}})(\tfrac{{\partial }^{3}w}{\partial {x}^{3}}+\tfrac{{\partial }^{3}w}{\partial x\partial {y}^{2}})\\ \,\,\,\,+\,(\tfrac{C}{2}){(\tfrac{\alpha }{{h}_{0}})}^{2}(\tfrac{{\partial }^{2}w}{\partial {x}^{2}}+\nu \tfrac{{\partial }^{2}w}{\partial {y}^{2}})=\tfrac{f(x,y,t)}{(\rho {h}_{0})(1+\alpha \tfrac{x}{{h}_{0}})}\end{array}$$

where $$C=\tfrac{E{h}_{0}^{2}}{\rho (1-{\nu }^{2})}$$

- *ρ* = density of plate material (kg/m^3^).

Equation (5) is a 4^th^-order parabolic partial differential equation with variable coefficients. The analytical solution of Equation (5) using the homotopy perturbation method^11^ for the thin plate which is simply supported on all sides is given by:

6 $$w(x,y,t)=\sum _{m=1}^{\infty }\sum _{n=1}^{\infty }{w}_{mn}$$

6a $$\begin{array}{rcl}{\rm{where}}\,{w}_{mn} & = & \sum _{q=0}^{\infty }\,\tfrac{1}{{(1+\alpha )}^{2q-1}}\{{A}_{q}{S}_{x}{S}_{y}+{B}_{q}{[\sin (\tfrac{m\pi x}{a})]}^{\beta }{C}_{x}{S}_{y}\}\,\{{A}_{mn}\tfrac{{[{\lambda }_{mn}t]}^{2q}}{(2q)!}\\  &  & +\,(\tfrac{{C}_{mn}}{{\lambda }_{mn}})\tfrac{{[{\lambda }_{mn}t]}^{2q+1}}{(2q+1)!}\}\end{array}$$

7a7b7c $${S}_{x}=\,\sin (\frac{m\pi x}{a}),\,{S}_{y}=\,\sin (\frac{n\pi y}{b}),\,{C}_{x}=\,\cos (\frac{m\pi x}{a})$$

and $$\beta =\{\begin{array}{ll}1 & {\rm{on}}\,{\rm{the}}\,{\rm{boundary}}\\ 0 & {\rm{inside}}\,{\rm{the}}\,{\rm{boundary}}\end{array}$$

The natural frequency of the plate *λ*_*mn*_ is given by:

8a $${\lambda }_{mn}=(1+\alpha )(1+\alpha \frac{x}{{h}_{0}}){(\frac{{D}_{0}}{\rho {h}_{0}})}^{\frac{1}{2}}[{(\frac{m\pi }{a})}^{2}+{(\frac{n\pi }{b})}^{2}]$$

8b $$=(1+\alpha )(1+\alpha \frac{x}{{h}_{0}})\,{{\Omega }}_{mn}$$

9 $${\rm{where}}\,{{\Omega }}_{mn}={(\frac{{D}_{0}}{\rho {h}_{0}})}^{\frac{1}{2}}{\pi }^{2}[{(\frac{m}{a})}^{2}+{(\frac{n}{b})}^{2}]\,$$

for *m* = 1, 2, 3, … etc. and *n* = 1, 2, 3, … etc.

For a uniform thickness plate, α = 0, and Equation (8b) is identical to Equation (9) and

10 $${A}_{0}=1,\,{B}_{0}=0;\,{A}_{1}=-\,1,\,{B}_{1}=0;\,{A}_{2}=1,\,{B}_{2}=0$$

For a non-uniform plate, we have:

11a11b $${A}_{0}=1,\,\,{B}_{0}=0$$

12a $${A}_{1}=-\,1+(\frac{6}{{\lambda }_{mn}^{2}})(\frac{{D}_{0}}{\rho {h}_{0}}){(\frac{\alpha }{{h}_{0}})}^{2}[{(\frac{m\pi }{a})}^{2}+\nu {(\frac{n\pi }{b})}^{2}]$$

12b $${B}_{1}=(\frac{6}{{\lambda }_{mn}}){(\frac{{D}_{0}}{\rho {h}_{0}})}^{\frac{1}{2}}(\frac{\alpha }{{h}_{0}})(\frac{m\pi }{a})$$

13a $$\begin{array}{rcl}{A}_{2} & = & -\tfrac{1}{{\lambda }_{mn}^{2}}\{[{f}_{1}+40(\tfrac{{D}_{0}}{\rho {h}_{0}}){(\tfrac{\alpha }{{h}_{0}})}^{2}{(\tfrac{m\pi }{a})}^{2}]\\  &  & +6(\tfrac{\alpha }{{h}_{0}})(\tfrac{{{\Omega }}_{mn}}{{\lambda }_{mn}})[2(\tfrac{{D}_{0}}{\rho {h}_{0}})(1+\alpha \tfrac{x}{{h}_{0}})(\tfrac{\alpha }{{h}_{0}})[3{(\tfrac{m\pi }{a})}^{2}+{(\tfrac{n\pi }{b})}^{2}]\\  &  & +\,{f}_{3}(\tfrac{m\pi }{a})]+(\tfrac{6}{{\lambda }_{mn}^{2}})(\tfrac{{D}_{0}}{\rho {h}_{0}}){(\tfrac{\alpha }{{h}_{0}})}^{2}[{f}_{1}[{(\tfrac{m\pi }{a})}^{2}+\nu {(\tfrac{n\pi }{b})}^{2}]\\  &  & +2{(\tfrac{\alpha }{{h}_{0}})}^{2}(\tfrac{{D}_{0}}{\rho {h}_{0}}){[{(\tfrac{m\pi }{a})}^{2}+{(\tfrac{n\pi }{b})}^{2}]}^{2}\,[1+6{(\tfrac{m\pi }{a})}^{2}]]\}\end{array}$$

13b $$\begin{array}{rcl}{B}_{2} & = & -\tfrac{1}{{\lambda }_{mn}^{2}}\{[{f}_{3}+8(1+\alpha \tfrac{x}{{h}_{0}})(\tfrac{\alpha }{{h}_{0}})(\tfrac{m\pi }{a}){(\tfrac{{D}_{0}}{\rho {h}_{0}})}^{\tfrac{1}{2}}{{\Omega }}_{mn}]+6(1+\alpha \tfrac{x}{{h}_{0}})(\tfrac{\alpha }{{h}_{0}}){(\tfrac{{{\Omega }}_{mn}}{{\lambda }_{mn}})}^{2}\\  &  & \times \,[{f}_{1}+12(\tfrac{{D}_{0}}{\rho {h}_{0}}){(\tfrac{\alpha }{{h}_{0}})}^{2}[2{(\tfrac{m\pi }{a})}^{2}+{(\tfrac{n\pi }{b})}^{2}](\tfrac{m\pi }{a})]+(\tfrac{6}{{\lambda }_{mn}^{2}})(\tfrac{{D}_{0}}{\rho {h}_{0}}){(\tfrac{\alpha }{{h}_{0}})}^{2}\\  &  & \times \,[{f}_{3}[{(\tfrac{m\pi }{a})}^{2}+\nu {(\tfrac{n\pi }{b})}^{2}]+4(1+\alpha \tfrac{x}{{h}_{0}})(\tfrac{\alpha }{{h}_{0}})(\tfrac{m\pi }{a}){{{\Omega }}_{mn}}^{2}]\}\end{array}$$

14 $${f}_{1}(x)=-\,(\frac{C}{12}){(1+\alpha \frac{x}{{h}_{0}})}^{2}{[{(\frac{m\pi }{a})}^{2}+{(\frac{n\pi }{b})}^{2}]}^{2}+(\frac{C}{2}){(\frac{\alpha }{{h}_{0}})}^{2}[{(\frac{m\pi }{a})}^{2}+\nu {(\frac{n\pi }{b})}^{2}]$$

15 $${f}_{3}(x)=(\frac{C}{2})(1+\alpha \frac{x}{{h}_{0}})(\frac{\alpha }{{h}_{0}})[{(\frac{m\pi }{a})}^{3}+(\frac{m\pi }{a}){(\frac{n\pi }{b})}^{2}]$$

### Verification of results for frequency parameter

A non-dimensional frequency parameter^23^ can be defined for a rectangular plate as *λ*_*p*_ = *ka* where ${k}^{4}=(\frac{\rho h}{D}){\lambda }_{mn}^{2}$. Thus, *λ*_*p*_ can be expressed as:

16 $${\lambda }_{p}=\pi {(1+\alpha )}^{\frac{1}{2}}{[{m}^{2}+{n}^{2}{(\frac{a}{b})}^{2}]}^{\frac{1}{2}}$$

Results of *λ*_p_ for b/a = 1 are compared to those from existing literature^24,25^ in Table 2. Similarly, results for *b*/*a* = 2 are shown in Table 3. The present solution is denoted as PS-2. The frequency parameter computed from the analytical model compares favourably with data from existing literature with errors not exceeding 7%, thus verifying accuracy of the model.

**Table 2:** Frequency parameter, *λ*_*p*_ for *b*/*a* = 1, *ν* = 0.3.

mode	m	n	α = 0.1			% error of PS-2 rel. to ref.^24^
			ref.^24^	PS-2	ref.^25^	
1	1	1	4.660	4.660	4.661	0
2	1	2	7.362	7.368	—	0
3	2	1	7.363	7.368	—	0
4	2	2	9.311	9.319	—	0
5	1	3	10.389	10.419	—	0
6	3	1	10.393	10.419	—	0
7	2	3	11.850	11.880	—	0
8	3	2	11.852	11.88	—	0
9	1	4	13.496	13.585	—	0
10	4	1	13.659	13.585	—	0
11	3	3	13.730	13.979	—	1
12	2	4	14.628	14.735	—	0
13	4	2	14.631	14.735	—	0
14	3	4	16.358	16.475	—	0
15	4	3	16.360	16.475	—	0
16	1	5	16.451	16.801	—	2
17	5	1	16.468	16.801	—	2
18	2	5	17.411	17.744	—	1
19	5	2	17.416	17.744	—	1
20	4	4	18.469	18.639	—	0
21	3	5	18.88	19.213	—	1

**Table 3:** Frequency parameter, *λ*_*p*_ for *b*/*a* = 2, *ν* = 0.3.

mode	m	n	α = 0.1			% error of PS-2 rel. to ref.^24^
			ref.^24^	PS-2	ref.^25^	
1	1	1	3.684	3.684	3.684	0
2	1	2	4.659	4.660	—	0
3	1	3	5.930	5.940	—	0
4	2	1	6.789	6.793	—	0
5	2	2	7.322	7.368	—	0
6	1	4	7.362	7.368	—	0
7	2	3	8.226	8.237	—	0
8	1	5	8.903	8.872	—	0
9	2	4	9.091	9.319	—	2
10	3	1	9.995	10.021	—	0
11	3	2	9.977	10.419	—	4
12	1	6	10.393	10.419	—	0
13	2	5	10.405	10.549	—	1
14	3	3	10.853	11.052	—	1
15	2	6	11.021	11.880	—	7
16	3	4	11.460	11.880	—	3
17	1	7	11.826	11.997	—	1
18	3	5	12.728	12.867	—	1
19	2	7	13.177	13.282	—	0
20	4	1	13.479	13.585	—	0
21	1	8	13.967	13.585	—	−2

### Validation of analytical model

An irregular-shaped plate of variable thickness and with the same dimensions as that of Fig. 1 was designed using ANSYS. The initial and final thicknesses and the taper angle α are defined below Table 1. This plate is used for validating the analytical model. Table 1 shows a comparison of numerical (ANSYS) results of natural frequencies of the irregular-shaped plate of variable thickness with corresponding analytical results (PS-3) of a rectangular plate also of variable thickness. The boundary conditions used for numerical simulation allows movements in the x- and y- directions but not in the z-direction. These boundary conditions, which give results closest to those of simple supports are used in the numerical simulation as ANSYS does not support the simply supported condition for solid bodies^20^. The analytical model represented by the rectangular plate of variable thickness is simply supported on all sides.

**Figure 4:**
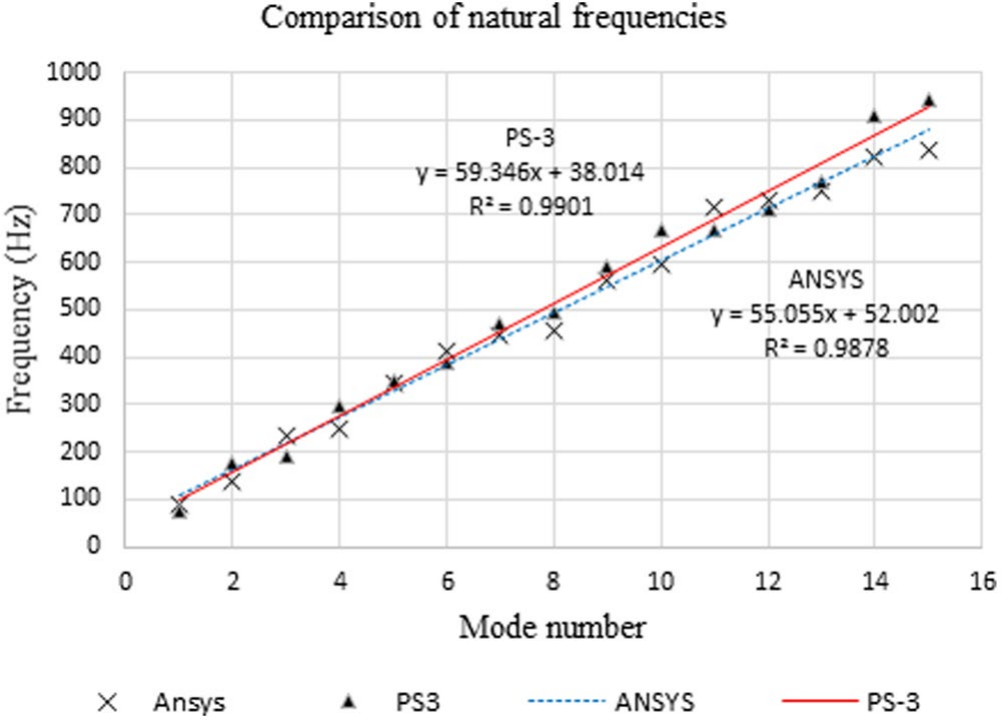
Comparison of natural frequencies. Analytical (**PS-3**)/Numerical (**ANSYS**).

The graphical results of Table 1 are shown in Fig. 4. Natural frequencies computed from this analytical model compare well with data from ANSYS with errors ranging from −18% to +18% except for the 2^nd^ mode. These results indicate good agreement between PS-3 and ANSYS thus validating the analytical model with variable thickness.

### Validation of sound power characteristics

The criteria for accurate simulation using finite element method is 6 acoustic elements per wavelength. The element size was thus chosen as 10 mm at a frequency of 5 kHz and speed of sound as 343.2 ms^−1^.

**Figure 5:**
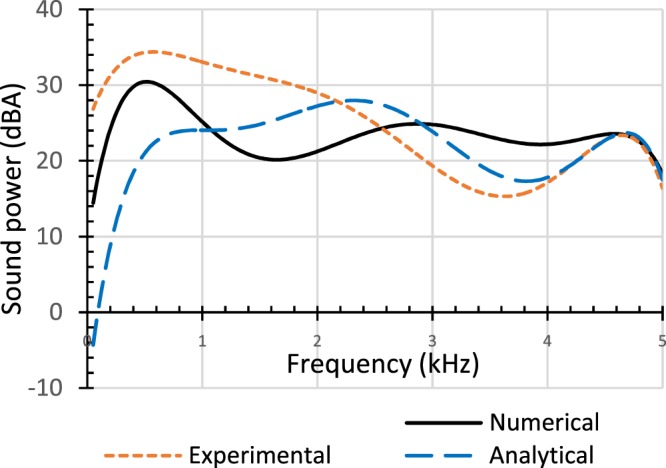
Validation of sound power results of the analytical model.

Sound power curves for White Spruce obtained from ANSYS and experiment for the variable thickness irregular-shaped plate of Fig. 1 and from computation using the analytical model are shown in Fig. 5. From around 200 Hz to 400 Hz, all three curves are similar in shape. The graph from the analytical model is similar in shape to that from experiment for frequencies around 2.4 kHz to 5 kHz and exhibits a minimum around 3.8 kHz. The minimum from experiment is around 3.6 kHz. This difference is to be expected as there is no sound hole in the case of the analytical model. The shapes of the graphs from the analytical model and from numerical analysis using ANSYS are similar from 1.5 kHz to 2.2 kHz. From 4.2 kHz to 5 kHz, the graphs for the analytical model and experiment are almost identical. All three graphs are almost identical from 4.7 kHz to 5 kHz. This shows that the analytical model can be used to compute the sound power of an irregular-shaped plate.

### Comparison of natural frequencies

As the simply supported condition is not supported in ANSYS, this boundary condition was therefore simulated numerically using “displacement” boundary condition, i.e. the irregular-shaped plate is allowed translation in the *x*- and *y*- directions but fully constrained in the *z*-direction. The numerical results in Fig. 4 indicate good agreement with those of PS-3 to within 18% except for mode 2. At lower frequencies, the simulated support behaves like a simple support while the behaviour varies between partially free and simple support at higher frequencies. The effect of material property such as viscoelastic damping of the material on the natural frequencies can be considered negligible in comparison with the effect of the simulated support which is physical in nature. The implication of the simulated support is that it allows unlimited motion of the irregular-shaped plate in the x- and y-directions compared to the simple support of the analytical model and hence affects the magnitudes of the natural frequencies for higher modes. It is worthy to note that control of natural frequencies is also possible with the use of scalloped braces^6,26,27^.

### Comparison of sound power

The graph of Fig. 5 obtained from simulating the transverse vibration of the unbraced variable thickness irregular-shaped plate using ANSYS shows a minimum at around 1.5 kHz whereas results from experiment for an identical plate shows a decrease in sound power from about 600 Hz to 2 kHz and a steeper decrease to a minimum around 3.6 kHz. This indicates a progressive transfer of sound power from the sound hole to the plate in both cases. This transfer of sound power is more gradual in the experiment than in the numerical simulation. Both curves show that sound power increases again after the minima before finally dropping to a minimum at 5 kHz. The shapes of these sound power curves for soundboards are similar to those obtained experimentally^28^ from 100 Hz to 600 Hz for traditional (1 hole at the top), Kasha (1 hole each at the top and side) and NFH (no hole at the top but 2 holes at the side) guitars. Higher sound power from these guitars resulted from the use of amplified white noise to vibrate their stiffer soundboards due to bracings. The magnitude of sound power in Fig. 5 is a result of applying a sinusoidal force of 0.1 N near the sound hole of the unbraced plate in simulation and experiment. The same force is applied near the mid-point of the analytical model. Results of sound power for the analytical model are validated experimentally and numerically. Thus, it is possible to compute the sound power of an irregular-shaped plate by using the analytical model.

### Summary

The analytical model of the irregular-shaped plate is an equivalent thin rectangular plate of variable thickness which is simply supported on all sides. Criteria for determining its dimensions are presented. “Displacement” boundary condition is used to simulate the simply supported condition in ANSYS as well as implemented experimentally. Results of sound power computed from the analytical model are validated experimentally and numerically using an irregular-shaped plate with dimensions identical to that of a Torres’ model of a soundboard. It is shown that the analytical model with variable thickness can be used to compute the sound power of unbraced irregular-shaped plates.

## Methods

An analytical model is used to compute the sound power of an irregular-shaped variable thickness plate defined by the parameter α. Mathematically, this implies solving the 4^th^ order parabolic partial differential equation of motion of a variable thickness rectangular plate and computing its sound power using the analytical solution.

### Experimental investigation of sound power of irregular-shaped plate of variable thickness

**Figure 6:**
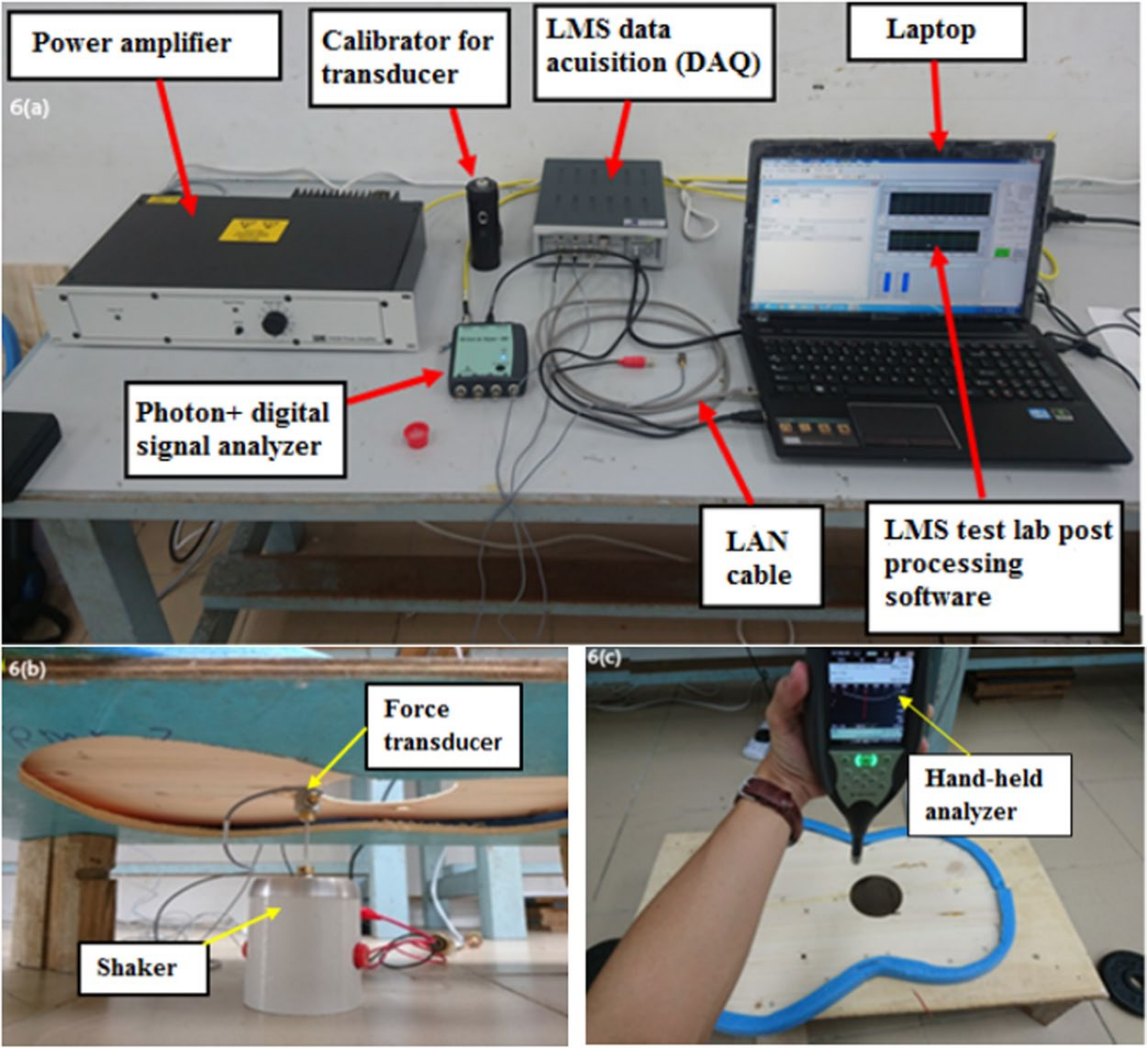
Instrumentation for sound power measurement. (**a**) Arrangement of equipment (**b**) The shaker (B&K Model Y201. M4-CE, Series No: 400437-6) and force transducer (**c**) Hand-held analyser (B&K type 2250).

The experimental setup is shown in Fig. 6(a). A B&K shaker was used to provide a sinusoidal input to the plate through a force transducer shown in Fig. 6(b).

Sound power was measured *in situ* in accordance with ISO 9614-1:1993^29^ which requires sound intensity to be measured at discrete points normal to a virtual surface. A B&K hand-held analyzer type 2250 sound level meter fitted with sound intensity software BZ-7233 was used to measure sound intensity around a discrete point 30 cm from the top surface around the sound hole of the plate as shown in Fig. 6(c). A ½-inch Type 4190 free-field microphone attached to the hand-held analyzer was used to record sound power over a portion of the plate around the discrete point. The unit of sound power is dBA. The results of sound power up to 5 kHz were saved to Microsoft Excel for customised reporting and further post-processing. These results are tabulated in Table B of Appendix B under “Supplementary Information”. The energy of vibration of a simply supported rectangular plate of uniform thickness is given by^30^ and its extension to rectangular plates of variable thickness is provided in Appendix A1 of “Supplementary Information”. 

### Analytical computation of sound power

The simply-supported variable thickness rectangular plate has a variable thickness given by Equation (3) and a mode shape given by Equation (6a). It has dimensions of *a* = 374.3 mm and *b* = 350.0 mm in the *x*- and *y*-directions respectively. This plate is excited by a harmonic point force of amplitude |F| of 0.1 N at location (*x*_0_, *y*_0_) = (187, 175) mm. Its sound power, *W* derived in Appendix A1 of “Supplementary Information” is shown in Equation (17) and computed using a MATLAB program.

17 $$W=\frac{2\rho Cab{|{\rm{F}}|}^{2}{(1+\alpha )}^{2}}{{M}^{2}}\sum _{m=1}^{\infty }\sum _{n=1}^{\infty }{\sigma }_{mn}\frac{{si}{{n}}^{2}(\frac{m\pi {x}_{0}}{a}){\sin }^{2}(\frac{n\pi {y}_{0}}{b}){\omega }_{mn}^{2}}{[{({\omega }_{mn}^{2}-{\omega }^{2})}^{2}+{\omega }_{mn}^{4}{\eta }^{2}]}$$

where *M* = mass of plate = 0.143 kg,

α = 0.003,

*η* = structural damping ratio = 0.01,

*ω*_*mn*_ = natural frequency of mode (*m*, *n*) from Equation (8a),

*ω* = excitation (rad/s),

*σ*_*mn*_ = radiation efficiency of mode (*m, n*),

*ρ* = density of plate = 430 kg/m^3^ for White Spruce,

*C* = 343.2 m/s (velocity of sound in air at 20 °C).

An expression for the radiation efficiency *σ*_*mn*_ is derived as shown in Appendix A2 of “Supplementary Information”. Sound power is computed for a total of 25 lowest odd-odd modes using a MATLAB program by adding sound power for values of *m* = 1, 3, 5, …, 9 and *n* = 1, 3, 5, …, 9.

### Numerical analysis

It was intended to use the simply-supported boundary conditions but since ANSYS does not support this condition for solid bodies^20^, it was decided to simulate this condition using displacement boundary condition by allowing freedom of movement in the *x*- and *y*-directions but fixed in the *z*-direction.

Numerical results of sound power over the entire top surface of the irregular-shaped plate are simulated using ANSYS (Release18). Velocities in the *z*-direction normal to the plate surface from the harmonic structural module are used as acoustic excitation sources in the harmonic acoustics module. The structure is then suppressed in the acoustic module (no structure-fluid interaction) resulting in an uncoupled acoustic analysis in solving for the sound power. All values of sound power are scaled by a factor of 35% and tabulated in Table B of Appendix B. This is an assumption that one microphone can only map 35% of the total top surface area of the plate.

Table B also shows the experimental data for sound power for the unbraced variable thickness plate fabricated from White Spruce as the wood type. This plate was machined using a CNC machine and does not have any bracing and bridge.

The analytical model in predicting sound power of the variable thickness plate is thus validated experimentally and numerically using ANSYS. However, as shown in Appendix A2, the expression for the radiation efficiency σ_*mn*_ given by Equation (A2.2) is valid for values of R → 0 and low values of ω_*mn*_. This is a limitation of the present analytical model.

The thickness of the irregular-shaped plate used in numerical analysis (**ANSYS**) varies from 2.0 mm at the lower bout to 3.297 mm at the upper bout. Length of plate is 482.6 mm, thus resulting in a taper angle, α = 0.154 degrees.

*Results are computed from those obtained for the uniform thickness plate where the natural frequency of the variable thickness plate is given by the minimum value of *λ*_*mn*_ from Equation (8a). The minimum value of Equation (8a) is given by:

16a $${\lambda }_{mn}=(1+\alpha ){(\frac{{D}_{0}}{\rho {h}_{0}})}^{\frac{1}{2}}[{(\frac{m\pi }{a})}^{2}+{(\frac{n\pi }{b})}^{2}]\,{\rm{with}}\,x={\rm{0}}$$

16b $$=\,(1+\alpha ){{\Omega }}_{mn}$$

where *α* = 0.003 radians and *Ω*_*mn*_ is given by Equation (9).

## Electronic supplementary material

Supplementary Information
