# Supplementary material for: An analytical model for computing the sound power of an unbraced irregular-shaped plate of variable thickness
Source: Sci Rep. 2018 Oct 18;8:15355. doi: 10.1038/s41598-018-33645-y (PMC6194024; doi:10.1038/s41598-018-33645-y)
Supplement: Supplementary file 1 — Supplementary Information [file 41598_2018_33645_MOESM1_ESM.pdf]

Supplementary Information

An analytical model for computing the sound power of an unbraced irregular-shaped plate of variable thickness

[1]MENG KOON LEE

[2]MOHAMMAD HOSSEINI FOULADI

[3]SATESH NARAYANA NAMASIVAYAM

School of Engineering, Taylor's University

No.1 Jalan Taylor's, 47500, Subang Jaya, Selangor, Malaysia

[1]leemengkoon@sd.taylors.edu.my

*[2]mfoolady@gmail.com

[3]SateshNarayana.Namasivayam@taylors.edu.my

[*]corresponding author

Appendix A1: Analytical expression for sound power. The energy of vibration of a simply-supported uniform thickness rectangular structure excited by a harmonic point force $|F|$ at a point (x_0, y_0)[30] is given by:

$$E(\omega) = \frac{2}{M}\sum_{m=1}^{\infty}\sum_{n=1}^{\infty}|F|^2 \frac{sin^2\left(\frac{m\pi x_0}{a}\right)\sin^2\left(\frac{n\pi y_0}{b}\right)\omega_{mn}^2}{\left[\left(\omega_{mn}^2-\omega^2\right)^2+\omega_{mn}^4\eta^2\right]} \quad \text{(A1.1)}$$

$$= \sum_{m=1}^{\infty}\sum_{n=1}^{\infty} E_{mn}$$

where M = the mass of the plate, and

$$E_{mn} = \frac{2}{M}|F|^2 \frac{sin^2\left(\frac{m\pi x_0}{a}\right)\sin^2\left(\frac{n\pi y_0}{b}\right)\omega_{mn}^2}{\left[\left(\omega_{mn}^2-\omega^2\right)^2+\omega_{mn}^4\eta^2\right]} \quad \text{(A1.1a)}$$

The space averaged mean square velocity, $< v^2 >$ is related to this energy by:

$$< v^2 >= \sum_{m=1}^{\infty}\sum_{n=1}^{\infty} < v_{mn}^2 > = \sum_{m=1}^{\infty}\sum_{n=1}^{\infty}\frac{E_{mn}}{M} \quad \text{(A1.2)}$$

where $< v_{mn}^2 >= \frac{E_{mn}}{M}$

The total sound power *W*, is given by:

$$W = \rho CS \sum_{m=1}^{\infty}\sum_{n=1}^{\infty}\sigma_{mn} < v_{mn}^2 > \quad \text{(A1.3)}$$

where ρ = density of plate,

C = velocity of sound in air,

S = surface area of plate,

σ = radiation efficiency of plate.

Combining Equations (A1.1), (A1.2) and (A1.3) gives:

$$W = \frac{2\rho Cab|\mathrm{F}|^2}{M^2}\sum_{m=1}^{\infty}\sum_{n=1}^{\infty}\sigma_{mn} \frac{sin^2\left(\frac{m\pi x_0}{a}\right)\sin^2\left(\frac{n\pi y_0}{b}\right)\omega_{mn}^2}{\left[\left(\omega_{mn}^2-\omega^2\right)^2+\omega_{mn}^4\eta^2\right]} \quad \text{(A1.4)}$$

for a rectangular plate with dimensions *a* and *b* in the *x*- and *y*-directions respectively.

Modification of Equation (A1.4) to include variable thickness plate

The general solution for the transverse displacement of a constant thickness plate is given in Lee *et al.*[11] by:

$w(x,y,t) = \sum_{m=1}^{\infty}\sum_{n=1}^{\infty}\left(\sin\frac{m\pi x}{a}\right)\left(\sin\frac{n\pi y}{b}\right)\left[A_{mn}\cos\Omega_{mn}t + \frac{C_{mn}}{\Omega_{mn}}\sin\Omega_{mn}t\right]$. For sound power calculation, only the spatial part of $w(x,y,t)$ is required. Therefore:

$$w(x,y) = \sum_{m-1}^{\infty}\sum_{n=1}^{\infty}\left(sin\frac{m\pi x}{a}\right)\left(sin\frac{n\pi y}{b}\right) \quad \text{(A1.5)}$$

For a variable thickness plate, the general solution for a simply supported plate as given by Equation (6a) is:

$$w(x,y,t) = \sum_{m=1}^{\infty}\sum_{n=1}^{\infty}\sum_{q=0}^{\infty} v_q = \sum_{m=1}^{\infty}\sum_{n=1}^{\infty}\sum_{q=0}^{\infty}\frac{1}{(1+\alpha)^{2q-1}}\left\{A_q S_x S_y + B_q\left[\sin\left(\frac{m\pi x}{a}\right)\right]^{\beta} C_x S_y\right\}\left\{A_{mn}\frac{[\lambda_{mn}t]^{2q}}{(2q)!} + \left(\frac{C_{mn}}{\lambda_{mn}}\right)\frac{[\lambda_{mn}t]^{2q+1}}{(2q+1)!}\right\}$$

As a first approximation, considering only the lowest order term with $q = 0$ and for sound power calculation using only the spatial part of $w(x,y,t)$, we have:

$$w(x,y) = \sum_{m=1}^{\infty}\sum_{n=1}^{\infty}(1+a)\left\{S_x S_y\right\} \quad \text{(A1.6)}$$

where $A_0 = 1,\ B_0 = 0,\ S_x = sin\frac{m\pi x}{a},\ S_y = sin\frac{n\pi y}{b}$

Substituting the spatial part of the mode shape given by Equation (A1.6) into Equation (A1.4) gives the sound power of a variable thickness plate as:

$$W = \frac{2\rho Cab|\mathrm{F}|^2(1+\alpha)^2}{M^2}\sum_{m=1}^{\infty}\sum_{n=1}^{\infty}\sigma_{mn}\frac{sin^2\left(\frac{m\pi x_0}{a}\right)\sin^2\left(\frac{n\pi y_0}{b}\right)\omega_{mn}^2}{\left[\left(\omega_{mn}^2-\omega^2\right)^2+\omega_{mn}^4\eta^2\right]} \quad \text{(A1.7)}$$

Appendix A2: Analytical expression for radiation efficiency. The radiation efficiency σ_{mn} of a simply-supported uniform rectangular structure[30] is given by:

$$\sigma_{mn} = \left(\frac{2\omega_{mn}}{\pi Cab}\right)\int_S\int_{S'}\sin\frac{m\pi x}{a}\sin\frac{n\pi y}{b}\frac{\sin kR}{R}\sin\frac{m\pi x'}{a}\sin\frac{n\pi y'}{b}\,dxdydx'dy' \quad \text{(A2.1)}$$

where $k = \frac{\omega_{mn}}{C}$,

ω_{mn} = eigenfrequency of mode (m,n),

C = velocity of sound in air at 20^0 C= 343.2 ms^{-1},

and R is the distance between observation point (x,y) in air and point (x',y') on the surface of the plate given by:

$$R = \{(x - x')^2 + (y - y')^2\}^{\frac{1}{2}} \tag{A2.2}$$

For measurement of acoustic power[30], we have for $R \to 0$, $\frac{\sin kR}{R} \to k = \frac{\omega_{mn}}{C}$

$$\text{Now, } \int_0^a \sin\frac{m\pi x}{a}\,dx = \begin{cases} \left(\frac{2a}{m\pi}\right) & \text{for } m = 1,3,5, \dots \text{ etc} \\ 0 & \text{for } m = 2,4,6, \dots \text{ etc} \end{cases}$$

$$\text{Similarly, } \int_0^b \sin\frac{n\pi y}{b}\,dx = \begin{cases} \left(\frac{2b}{n\pi}\right) & \text{for } n = 1,3,5, \dots \text{ etc} \\ 0 & \text{for } n = 2,4,6, \dots \text{ etc} \end{cases}$$

However, for a simply-supported thin rectangular plate of variable thickness, the shape function is as given in Equation (A1.5).

Therefore Equation (A2.1) can be shown to be given by:

$$\sigma_{mn} = \left(\frac{32\omega_{mn}^2 ab}{\pi^5 m^2 n^2 C^2}\right)(1 + \alpha)^2 \tag{A2.3}$$

Appendix B: Sound power of unbraced irregular-shaped variable thickness plate for White Spruce.

Only 1 microphone was available for the experiment. Therefore, it was decided to determine a method for estimating the best results for Table B from experimental data. Eventually, it was decided that using a percentage of area of 35% mappable by 1 microphone in the experiment gives best results for Table B as any numerical value less than or greater than 35% resulted in higher errors. The estimation of percentage area covered by the same microphone at the same distance of 30 cm vertically above the equivalent rectangular plate is estimated as follows:

Estimation of percentage of area covered by 1 microphone for equivalent rectangular plate

The percentage of area mapped by the same microphone and at the same distance of 30 cm vertically above the rectangular plate is computed as follows:

Assumption: Area mapped by 1 microphone in experiment = 35% of total top surface area of irregular-shaped plate.

Let S_0 = total top surface area of irregular-shaped plate,

S_m = top surface area mapped by 1 microphone in experiment,

S_p = top surface area of rectangular plate without hole,

x = percentage of top surface area of equivalent plate mapped by the same microphone.

From experiment, we have:

$$\frac{S_m}{S_0} = 0.35 \tag{B1.1}$$

For rectangular plate, we have:

$$\frac{S_m}{S_p} = x \tag{B1.2}$$

Dividing Equation (B1.2) by Equation (B1.1) and simplifying gives:

$$x = 0.35\left(\frac{S_0}{S_p}\right) \tag{B1.3}$$

From Figure 1(a), area of the plates, s_0 = 0.125 m^2 and s_p = 0.374 x 0.350 = 0.131 m^2. From Equation (B1.3), we get:

$$x = 0.35\left(\frac{0.125}{0.131}\right) = 0.334 = 33.4\% \tag{B1.4}$$

Let I_0 = sound intensity generated by irregular-shaped plate in experiments,

I_1 = sound intensity generated by equivalent rectangular plate.

Multiplying the numerator and denominator of Equation (B1.1) and Equation (B1.2) by I_0 and I_1 respectively gives:

$$\frac{I_0 S_m}{I_0 S_0} = 0.35 \tag{B1.5}$$

$$\frac{I_1 S_m}{I_1 S_p} = x \tag{B1.6}$$

Eq. (B1.5) shows that the sound power captured by the microphone is 35% of the total sound power generated by the irregular-shaped plate. Likewise, Eq. (B1.6) shows that the sound power captured by the same microphone is $x = 33.4\%$ of the total sound power generated by the equivalent rectangular plate.

Mechanical and physical properties of White Spruce are:

a) Longitudinal Young's modulus (E): 10.0 GPa
b) Density (ρ): 430 kg/m^3
c) Poisson's ratio (ν): 0.43

Table B

Comparison of sound power

Plates without bracing (α=0.154 degrees)<br>Material: White Spruce				
Sound power (dBA)<br>(Ref: 1 x 10^{-12} watts)				
Set	Frequency (Hz)	**ANSYS** (R18)	Experiment (**Expt**)	Rectangular plate (374.3x350.0) mm
1	50.0	10.2	25.2	-6.3
2	100.0	22.4	31.2	2.5
3	150.0	24.6		
4	160.0		30.1	4.7
5	200.0	25.1	29.4	7.4
6	250.0		31.9	10.8
7	315.0		34.9	17.3
8	359.4	24.1		
9	400.0		35.2	30.7
10	500.0		32.4	15.3
11	630.0		35.3	18.3
12	668.8	28.4		
13	800.0		34	19.5
14	978.1	28.9		
15	1000.0		31.5	27.6
16	1250.0		31.6	25.9
17	1287.5	24		
18	1596.9	19.8		
19	1600.0		31.5	20.8
20	1906.2	12.7		
21	2000.0		31.5	36.7
22	2215.6	27.1		

23	2500.0		21.6	18.6
24	2525.0	24.4		
25	2834.4	29.5		
26	3143.8	18.4		
27	3150.0		19.3	26.1
28	3453.1	25.2		
29	3762.5	24.1		
30	4000.0		16.8	17.0
31	4071.9	18.8		
32	4381.2	25.5		
33	4690.6	23		
34	5000.0	18.2	16.4	17.6
